# Regulation of neuronal ankyrin localization and function by post-translational modifications

**DOI:** 10.1042/BST20253016

**Published:** 2025-05-01

**Authors:** Kalynn M. Bird, Paul M. Jenkins

**Affiliations:** 1 Department of Pharmacology, University of Michigan Medical School, Ann Arbor, MI, U.S.A; 2 Department of Psychiatry, University of Michigan Medical School, Ann Arbor, MI, U.S.A

**Keywords:** ankyrin, neurodevelopmental disorders, palmitoylation, phosphorylation, protein localization, ubiquitination, voltage-gated sodium channel

## Abstract

Ankyrins are a family of intracellular scaffolding proteins that control the subcellular localization of a host of critically important signaling proteins within neurons, including many proteins associated with neurological disease. Ankyrin proteins are a vital component of the neuron. These scaffolding proteins must be spatially and temporally arranged to interact with their binding partners and facilitate proper neuronal signaling. Dysfunction of ankyrins is associated with neurodevelopmental disorders such as epilepsy and autism spectrum disorder. Despite the high degree of sequence similarity between ankyrin proteins, they display almost completely nonoverlapping localization and function. How ankyrins localize to the correct subcellular compartments to interact with their binding partners and complete their distinct roles remains poorly understood. Emerging evidence suggests that post-translational modifications may play a key part in this process. Some of the post-translational modifications that have been identified to regulate ankyrins are phosphorylation, ubiquitination, and palmitoylation. These modifications affect proper interactions, function, and localization of ankyrin proteins, which highlights their potential role in disease. This review will give an overview of neuronal ankyrins, and how post-translational modifications could be utilized to regulate protein localization and function in the context of neurological disease.

## Ankyrin function in neurons

### Ankyrin structural and functional domains

Ankyrins are a family of scaffolding proteins that anchor a multitude of vital proteins including cell-adhesion molecules (CAMs), ion channels, and transporters to regulate excitable neuronal membranes. This scaffolding allows for proper localization of ankyrin-dependent binding partners which supports cell polarity and function [1–5]. Illustrating their significance, dysfunction of ankyrins is linked with a myriad of neurological diseases [6–11]. There are three ankyrin family members: ankyrin-R, ankyrin-B, and ankyrin-G, which are encoded by *ANK1*, *ANK2*, and *ANK3*, respectively. Ankyrin proteins are composed of multiple domains including the membrane-binding domain which contains 24 ankyrin repeats that serve as binding sites for its many partners [12,13]. Ankyrins tether their membrane-bound cargo to the underlying actin cytoskeleton by binding the actin-associated cytoskeletal proteins, β-spectrins, using the first of two Zu5 domains [12,14–16]. β-spectrin is a part of the spectrin heterotetrameric complex that binds to actin, which in turn allows ankyrin to tether its membrane cargo to the actin cytoskeleton [12,17,18]. Ankyrin proteins also contain a death domain that was named due to its similarity to a homologous domain in the Fas receptor which has a major role in triggering apoptosis [19]. Nonetheless, the function of the ankyrin death domain remains incompletely understood. Finally, ankyrins have a C-terminal domain which is the most variable region of the ankyrin protein and is primarily involved in mediating protein interactions and influencing protein localization through interaction with the membrane-binding domain [20–23].

### Ankyrin expression patterns

Despite their structure and sequence homology, each ankyrin protein has distinct expression patterns. Ankyrin-R was the first ankyrin protein to be cloned and is most highly characterized in erythrocytes [1]. However, recent work has shed light on additional roles for ankyrin-R in neurons [24]. Ankyrin-R is highly expressed in the cerebellum, where it is predominantly found in Purkinje neurons, granule cells, and cerebellar nuclei [25]. In the forebrain, ankyrin-R is enriched in parvalbumin-positive neurons, a subset of fast-spiking GABAergic interneurons that project onto pyramidal cells, where it regulates excitability through its interaction with Kv3.1b channels and maintenance of perineuronal nets [26]. The other two members of the ankyrin family, ankyrin-B and ankyrin-G, are highly expressed throughout the nervous system, where they play vital roles in multiple cell types, including neurons, oligodendrocytes, and astrocytes [27,28]. Highlighting their significance, ankyrin dysfunction is implicated in a multitude of neurological disorders [4,29,30].

### Ankyrin localization and function

**Figure 1:**
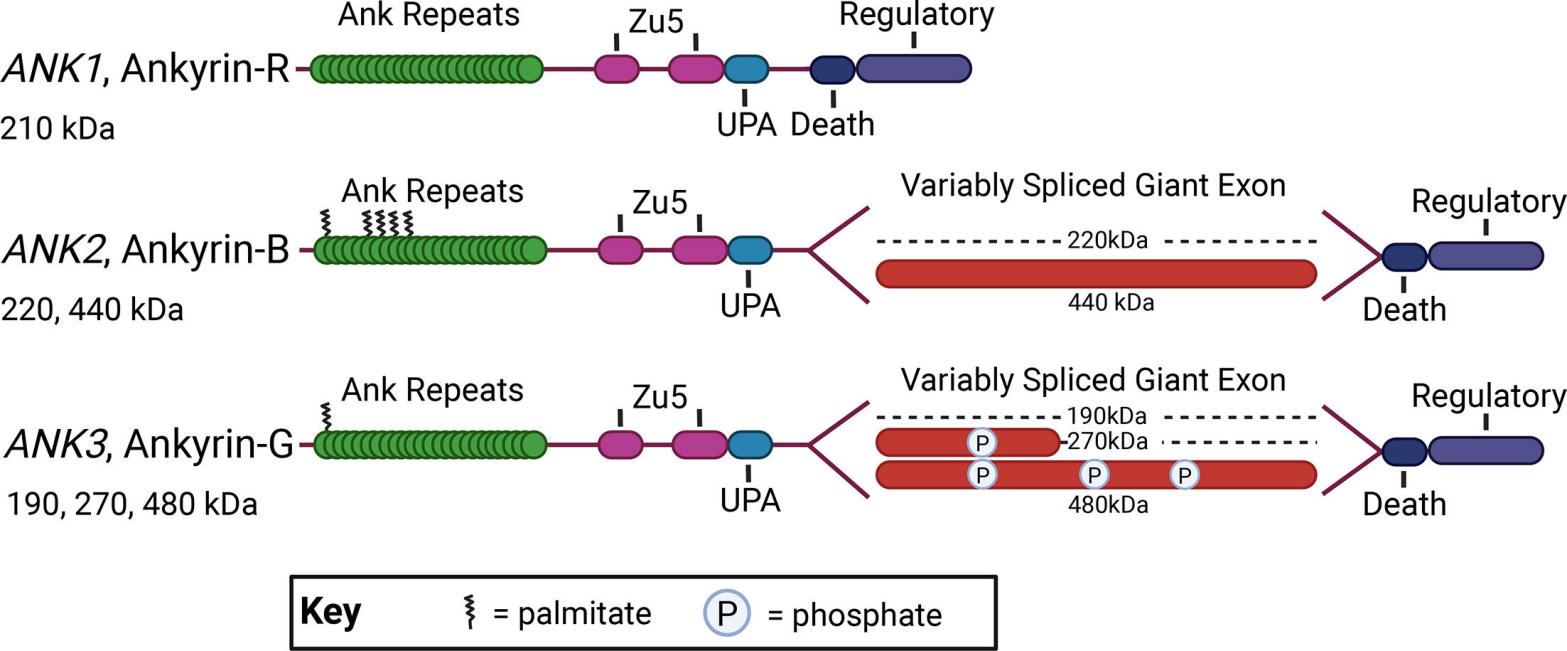
Ankyrin family of scaffolding proteins. Diagram of splice variants, key protein structural domains, and post-translational modifications of the ankyrin family of scaffolding proteins.

Within the neuron, ankyrins have specific subcellular localization that allows them to scaffold unique binding partners and regulate overall neuronal function. The genes encoding ankyrins are alternatively spliced which allows for each isoform to play diverse roles at different locations [31] (Figure 1). Alternative splicing of the *ANK3* gene gives rise to three main classes of ankyrin-G protein in the nervous system with sizes of 190, 270, and 480 kDa. Perhaps the most well-characterized function of ankyrin-G in neurons is the formation of the axon initial segment (AIS) by the 270 and 480 kDa isoforms [27,32]. The AIS is a specialized region located at the first 20–40 μm of the axon that is responsible for action potential initiation due to the voltage-gated sodium channels (VGSCs) concentrated there [32–36]. At the AIS, ankyrin-G targets ion channels, CAMs, and other cytoskeletal proteins in the membrane to regulate structural integrity, polarity, and excitability [32,37–40]. Beyond its role in AIS assembly, 480 kDa ankyrin-G is also present at the somatodendritic regions in mature neurons where it stabilizes GABAergic synapses [41]. The 190 kDa isoform of ankyrin-G plays a unique role in dendritic spines where it supports spine morphology and regulates glutamatergic synapses [42]. Thus, these proteins are involved in action potential initiation and propagation, as well as inhibitory and excitatory synaptic function due to the three unique ankyrin-G isoforms completing separate functions.

Ankyrin-B is similarly alternatively spliced to produce 220 and 440 kDa isoforms that also have separate localization and activity [43]. The 440 kDa variant of ankyrin-B is found at the unmyelinated axon where it regulates axonal branching through interaction with microtubules [44]. The 220 kDa ankyrin-B is present at the distal axon where it promotes axonal cargo transport [45]. Additionally, 220 kDa ankyrin-B is concentrated at the dendritic membrane where it recruits the VGSC Na_V_1.2, which mediates dendritic excitability and action potential backpropagation [46]. This isoform of ankyrin-B is also enriched at the cortical synapse where it regulates intracellular and surface levels of Ca_V_2.1, a channel that is essential for fast neurotransmitter release at presynaptic terminals [47,48]. Similar to ankyrin-G, the ankyrin-B isoforms carry out specialized roles. Overall, proper localization of each ankyrin isoform so that it can function appropriately and scaffold its binding partners is essential to promote normal neuronal function. With this, it is important to understand how ankyrins themselves are regulated.

### Sequence regulation of ankyrins

While ankyrins have high sequence similarity, certain portions of the ankyrin primary sequence differ and can contribute to their distinct neuronal localization. Ankyrin-G contains a giant exon that encodes both a serine-rich and tail domain that are not found in ankyrin-B [27,49]. Both of these domains contribute to restricting the 270 and 480 kDa ankyrin-G to the AIS and preventing lateral mobility [50]. Additionally, the 480 kDa ankyrin-G includes a unique portion of the giant exon which encodes a domain predominantly expressed in neurons, and this distinguishes it from all of the other isoforms. Only the 480 kDa ankyrin-G recruits βIV-spectrin to the AIS and is necessary for overall AIS assembly, suggesting that this domain is necessary for these processes [32,51]. Another thing that differentiates ankyrin-G and -B is a single divergent exon that encodes a linker peptide between the membrane-binding domain and Zu5 domains [52]. This ankyrin-B-specific linker was shown to be the reason why ankyrin-B is largely localized to intracellular compartments in cultured cells instead of plasma membrane bound like ankyrin-G [52]. However, in neurons, 220 kDa ankyrin-B is localized to the plasma membrane, which suggests that there are alternative mechanisms that can contribute to membrane localization. Despite what we have learned about the role of specific sequences within the ankyrin family and their role in controlling localization and function of these proteins, some important questions remain unanswered. It is still unclear how ankyrins localize to discrete subcellular compartments and interact with their different binding partners. Recent evidence suggests that post-translational modifications could regulate ankyrin protein interactions, localization, and function.

## Post-translational modification of ankyrins

### Regulation of ankyrin interactions by phosphorylation

**Figure 2:**
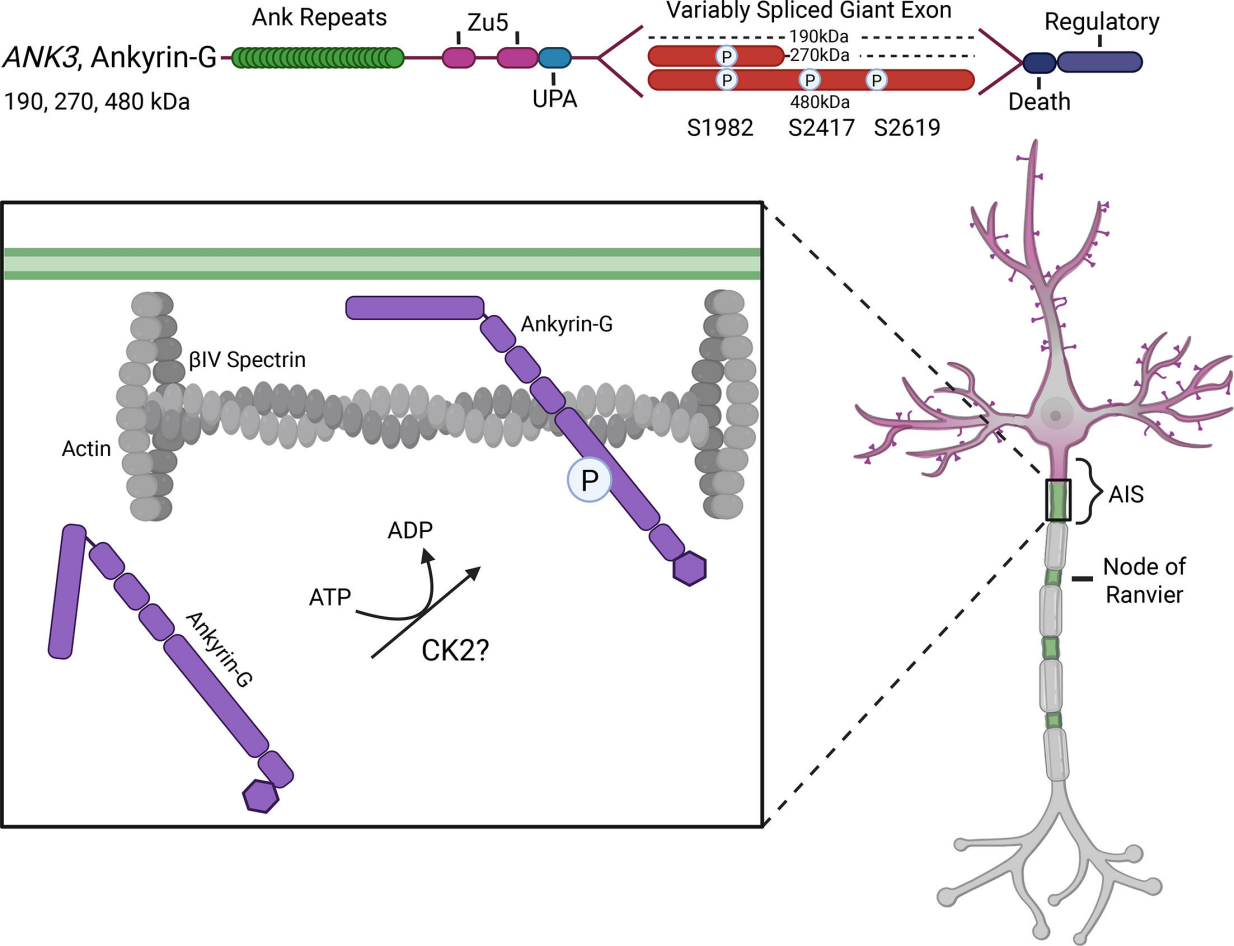
Ankyrin-G phosphorylation. (Top) Diagram of 480 kDa ankyrin-G highlighting the three identified sites of phosphorylation. (Bottom) 480 kDa ankyrin-G is phosphorylated to promote an open conformation that allows interaction with βIV-spectrin at the AIS.

Although many binding partners of ankyrins have been discovered, how and why ankyrins interact with their specific partners is less clear. Post-translational modifications such as phosphorylation have recently been shown to affect ankyrin scaffolding. Phosphorylation is the reversible addition of a phosphate group to a protein typically on serine, threonine, or tyrosine residues. The dynamic regulation of phosphorylation has a key role in many cellular processes and signaling through protein activation and inactivation [53,54]. The addition of the phosphate group to a protein is completed by kinases while dephosphorylation is mediated by phosphatases. Previous work has shown that phosphorylation of ankyrin-G affects its ability to interact with binding partners such as βIV-spectrin. Spectrin clustering at the AIS is critical for VGSC clustering and is dependent on its interaction with ankyrin-G at the first Zu5 domain [37,55,56]. Three sites of ankyrin-G phosphorylation have been recognized as significant for βIV-spectrin scaffolding at the AIS. S2417 in the 480 kDa ankyrin-G was one of the first sites identified [32]. The phospho-null mutant (S2717A) greatly reduced the ability of ankyrin-G to recruit βIV-spectrin but did not have an effect on ankyrin-G clustering at the AIS or in interacting with other binding partners [32]. S2417 is a site proposed to be phosphorylated by casein kinase 2 (CK2), which has previously been shown to regulate VGSC binding to ankyrin [57,58]. Two other sites were later identified as also disrupting ankyrin-G scaffolding of βIV-spectrin, S1982, and S2619 [51]. Unlike S2417A, mutation of these sites caused almost a complete lack of ankyrin-G recruitment of βIV-spectrin but also caused a decreased enrichment of ankyrin-G at the AIS, as well as a reduction in the amount of other ankyrin-G binding partners including neurofascin and VGSCs [51]. Interestingly, all of these identified sites are distant from the first Zu5 domain where ankyrin-G binds βIV-spectrin. This suggests that phosphorylation causes ankyrin-G to change from a closed conformation to an extended conformation that allows Zu5 domain availability for spectrin binding [51] (Figure 2). These data demonstrate the significance of phosphorylation on normal ankyrin-G function in spectrin scaffolding at the AIS.

Phosphorylation of ankyrin-B has been less studied, but likely occurs, and there have been several predicted phosphorylation sites determined through *in silico* work. In one study, 15 sites in the C-terminal domain have been identified as a possible ankyrin-B-specific localization mechanism [21]. However, these sites have not been confirmed experimentally, and the effects of ankyrin-B phosphorylation remain unresolved [21,59]. It is possible that phosphorylation at distinct sites may contribute to the differential binding and functions of these ankyrin proteins.

### Regulation of ankyrin localization by palmitoylation

Based on their structure, ankyrins would be predicted to be cytoplasmic, but it has been established that ankyrins are tightly membrane associated. One way soluble proteins can gain membrane association is through lipid modifications such as *S*-acylation, the reversible thioester linkage of fatty acid chains of varying length to a cysteine residue of a protein. One specific type of *S*-acylation is palmitoylation, which is the addition of the 16-carbon fatty acid palmitate. This modification regulates protein sorting and stability, as well as membrane trafficking [60]. The reversibility of palmitoylation is unique among lipid modifications and allows for intracellular shuttling of proteins to different membrane domains. Palmitoylation is performed by a family of enzymes called palmitoyl acyl transferases. These enzymes are diverse, but all contain a core zDHHC motif within the active site [61]. There have been ~23 mammalian zDHHC palmitoyl acyl transferases identified as responsible for palmitoylation [62]. How these enzymes recognize specific substrates is still an area of active research, as there is no known consensus sequence for palmitoylation. Tissue specificity and differential subcellular localization of palmitoyl acyl transferases are some ways in which these enzymes interact with different targets. Most zDHHC enzymes localize to the endoplasmic reticulum and Golgi apparatus, which means their substrates are often palmitoylated as they are synthesized and processed. However, there are some transferases at the plasma membrane which allow for local plasma membrane palmitoylation of substrates [63].

**Figure 3:**
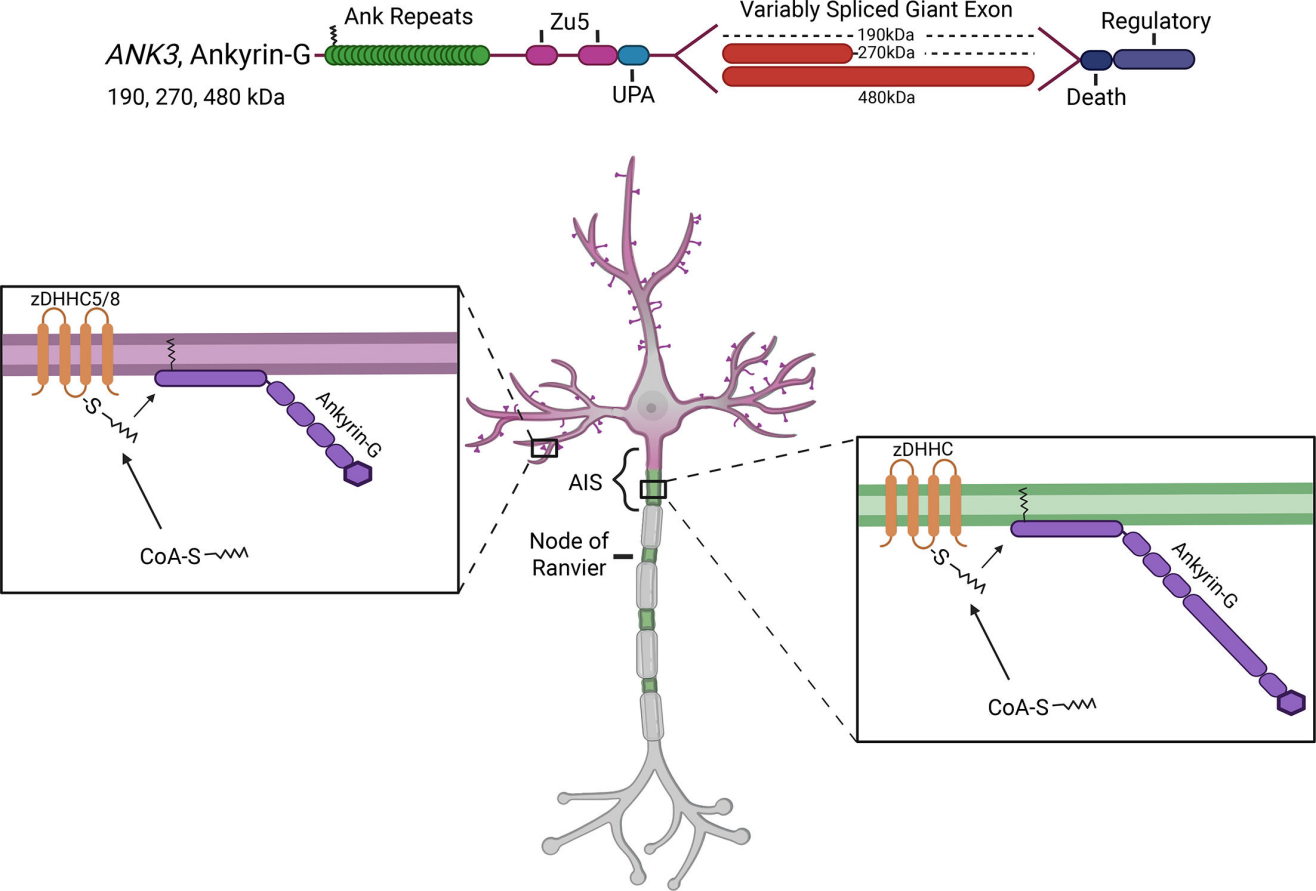
Ankyrin-G palmitoylation. (Top) Diagram of 190 and 480 kDa ankyrin-G highlighting the single site of palmitoylation. (Bottom left) 190 kDa ankyrin-G is palmitoylated to promote membrane association and localization at dendritic spines. (Bottom right) 480 kDa ankyrin-G is palmitoylated to promote membrane association and localization at the AIS.

Palmitoylation supports the organization of ankyrins at neuronal membranes and is necessary for ankyrin activity at these subcellular locations. Ankyrin-R was the first ankyrin family member found to be lipid modified, but little is known about how palmitoylation affects its localization and function [64]. Ankyrin-G was also found to be palmitoylated, and all tested functions of ankyrin-G thus far are reliant on its palmitoylation. The first characterization of ankyrin-G palmitoylation was using the 190 kDa isoform in polarized epithelial cells [65]. It was found that ankyrin-G is palmitoylated at a single cysteine residue (C70), and that this site is required for membrane association and function of ankyrin-G [65] (Figure 3). Similar results were seen with the C70A 270 and 480 kDa isoforms of ankyrin-G, which fail to cluster proteins at the AIS or stabilize GABAergic synapses on the soma and AIS [41,65]. Thus, all major isoforms of ankyrin-G are reliant on palmitoylation for their membrane association and function.

Two palmitoyl acyl transferases have been identified to be responsible for palmitoylation and membrane localization of ankyrin-G: zDHHC5 and -8 [66]. These two enzymes are closely related and exhibit functional redundancy in palmitoylation of ankyrin-G [66]. Unlike most palmitoyl acyl transferases, zDHHC5 and -8 localize to the plasma membrane [61]. In neurons, zDHHC5 is localized to the dendritic shaft, while zDHHC8 is targeted to the synapse [61,67]. Consistent with this, palmitoylation of ankyrin-G at C70 was also found to be important in the localization of 190 kDa ankyrin-G to dendritic spines [68]. Overexpression of C70A palmitoylation-dead 190 kDa ankyrin-G in cultured neurons causes changes in dendritic spine morphology and protein content [68]. This result is surprising considering endogenous ankyrin-G was present, suggesting that the palmitoylation-dead mutant of ankyrin-G is capable of acting as a dominant-negative. More recently, 480 kDa ankyrin-G was shown to be palmitoylated, and its palmitoylation is essential to its roles at the AIS and at GABA synapses, but it has not been confirmed whether this isoform is regulated by the same enzymes [41,69]. Although all of the ankyrin isoforms are palmitoylated at the same site, there is no known consensus sequence, and it is clear that sequences outside of the site of palmitoylation contribute to transferase specificity. Thus, while it is tempting to speculate that zDHHC5 and -8 palmitoylate 480 kDa ankyrin-G, it is possible that there are other unique enzymes for this ankyrin-G isoform.

**Figure 4:**
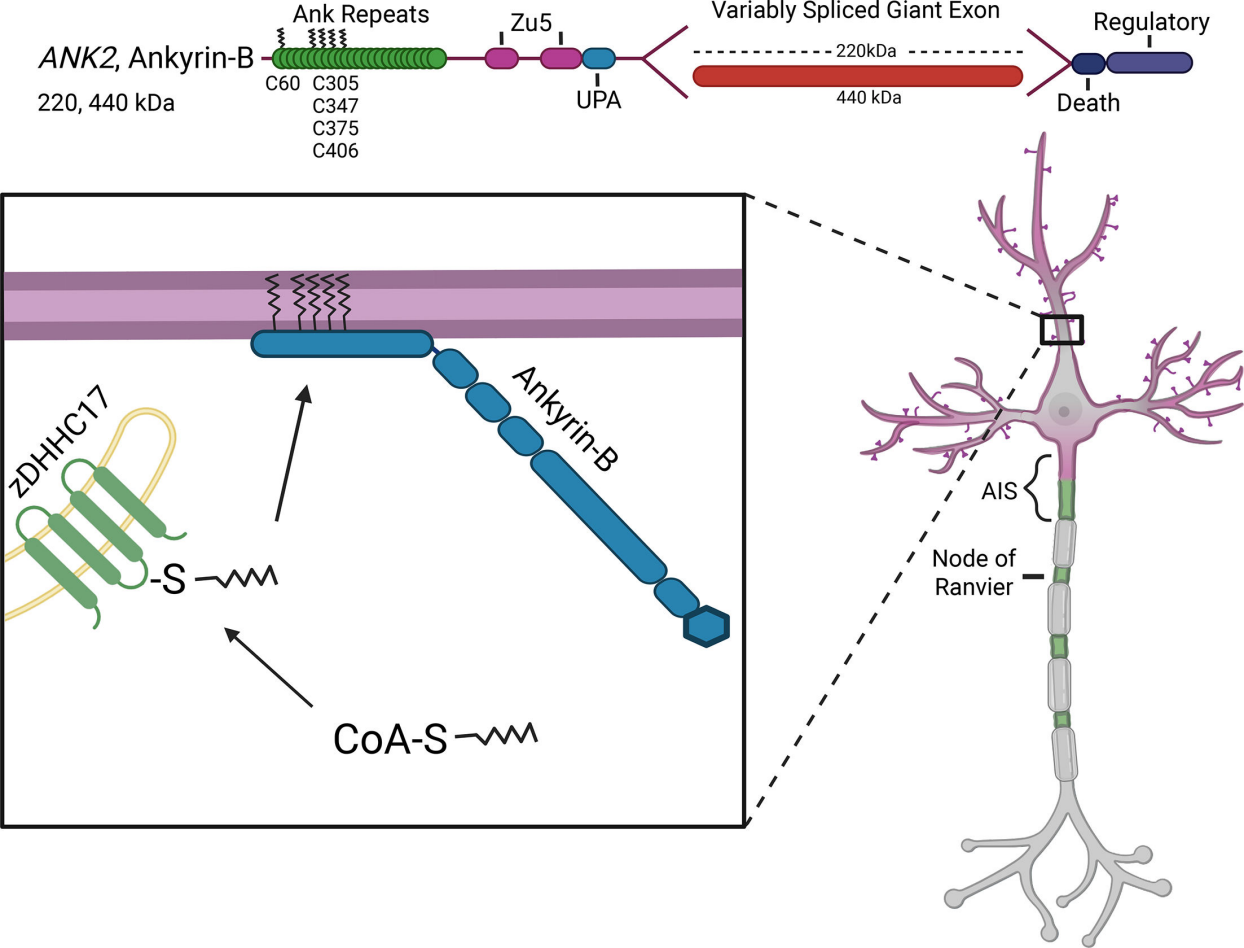
Ankyrin-B palmitoylation. (Top) Diagram of 220 kDa ankyrin-B highlighting five sites of palmitoylation. (Bottom) 220 kDa ankyrin-B is palmitoylated to promote membrane association and localization at dendrites.

Although ankyrins are highly homologous and even share a homologous cysteine to that which is palmitoylated in ankyrin-G, there are key differences between ankyrin-B and -G [69]. While ankyrin-B contains a homologous cysteine to C70 in ankyrin-G, it is not the only site of palmitoylation. Instead, five cysteines in ankyrin-B were found to be palmitoylated, and mutation of all five of these sites renders ankyrin-B palmitoylation-dead [69]. Ankyrin-B palmitoylation is necessary for its localization to dendrites, as well as its recruitment of the VGSC Na_V_1.2 (Figure 4) [69]. Although this mutant did not influence axonal cargo transport, it did prevent ankyrin-B localization to the dendritic membrane [69]. The lack of ankyrin-B at the dendrites prevents scaffolding of Na_V_1.2 and likely affects action potential backpropagation [69]. This illustrates both a palmitoylation-independent function of ankyrin-B in axonal transport and a palmitoylation-dependent function in Na_V_1.2 scaffolding at the dendrites. This is dissimilar to ankyrin-G where all of its functions tested thus far appear to depend on palmitoylation. Another disparity between ankyrin-B and -G palmitoylation is the palmitoyl acyl transferases responsible. zDHHC17 is the enzyme that palmitoylates ankyrin-B [69]. The presence of this enzyme is necessary for ankyrin-B palmitoylation and localization in neurons [69]. These results indicate that palmitoylation of each ankyrin at different sites and by different enzymes could contribute to how they achieve unique localization, binding partners, and functions at the membrane. It is also important to note that unlike ankyrin-G, the exact fatty acid chain length(s) added to ankyrin-B have not been confirmed, and this may be another mechanism of regulation.

Unlike most other lipid modifications, palmitoylation is reversible and dynamically regulated by depalmitoylating enzymes that hydrolyze thioester bonds between palmitate and substrate [70,71]. Depalmitoylation is much less understood compared with palmitoylation, in part because, like zDHHCs, how thioesterase enzymes identify their substrates remains unknown, and also because there is high variability in the dynamics of depalmitoylation across different proteins and palmitoylation sites [72,73]. Despite this, ankyrins are likely reversibly palmitoylated, as there exist multiple pools of ankyrin with palmitoylation-dependent and -independent functions and ankyrins exhibit developmental and therapeutic-induced changes in palmitoylation levels [68,69]. The enzymes that depalmitoylate ankyrins have not been established. Based on what is known currently, there are three classes of depalmitoylating enzymes: acyl protein thioesterases, alpha/beta hydrolase domain-containing 17 proteins, and palmitoyl protein thioesterases [70]. Palmitoyl protein thioesterase 1 (PPT1) is involved in the morphology and synaptic function of neurons [70]. One study screening for potential substrates of PPT1 found that ankyrin-G may be depalmitoylated by this enzyme [74]. With more work elucidating thioesterase enzymes, there will be a clearer picture of how ankyrin palmitoylation is enzymatically modulated which will allow for potential manipulation of the dynamic regulation of ankyrin localization.

### Regulation of ankyrin proteostasis and function by ubiquitination

There is a delicate balance of ankyrin expression levels so that proteostasis is maintained and there is no excessive protein aggregation [75]. A common method of regulating protein homeostasis is through degradation by the ubiquitin-proteasome system [76]. This system is dependent on the process of ubiquitination which is the addition of ubiquitin to a lysine residue of a protein. This process is catalyzed by an enzymatic cascade including E1, E2, and E3 enzymes. One feature of ubiquitin is that it can form polymeric chains that may serve as distinct cellular signals [77,78]. Broadly, ubiquitination of a protein marks it for degradation by the proteasome, but it can also regulate processes like protein trafficking [79]. Ankyrin-G is polyubiquitinated to promote degradation which regulates protein levels in the neuron [75]. The major neuronal E3 ligase Cdc20/APC is likely responsible for ankyrin-G polyubiquitination and subsequent degradation [75]. Previously, Cdc20/APC was shown to be crucial in dendrite morphogenesis [80]. Knockdown of Cdc20 in cultured neurons as well as *in vivo* caused impairment of dendrite formation and development [80]. This may be due to the disruption of ankyrin-G regulation by polyubiquitination.

**Figure 5:**
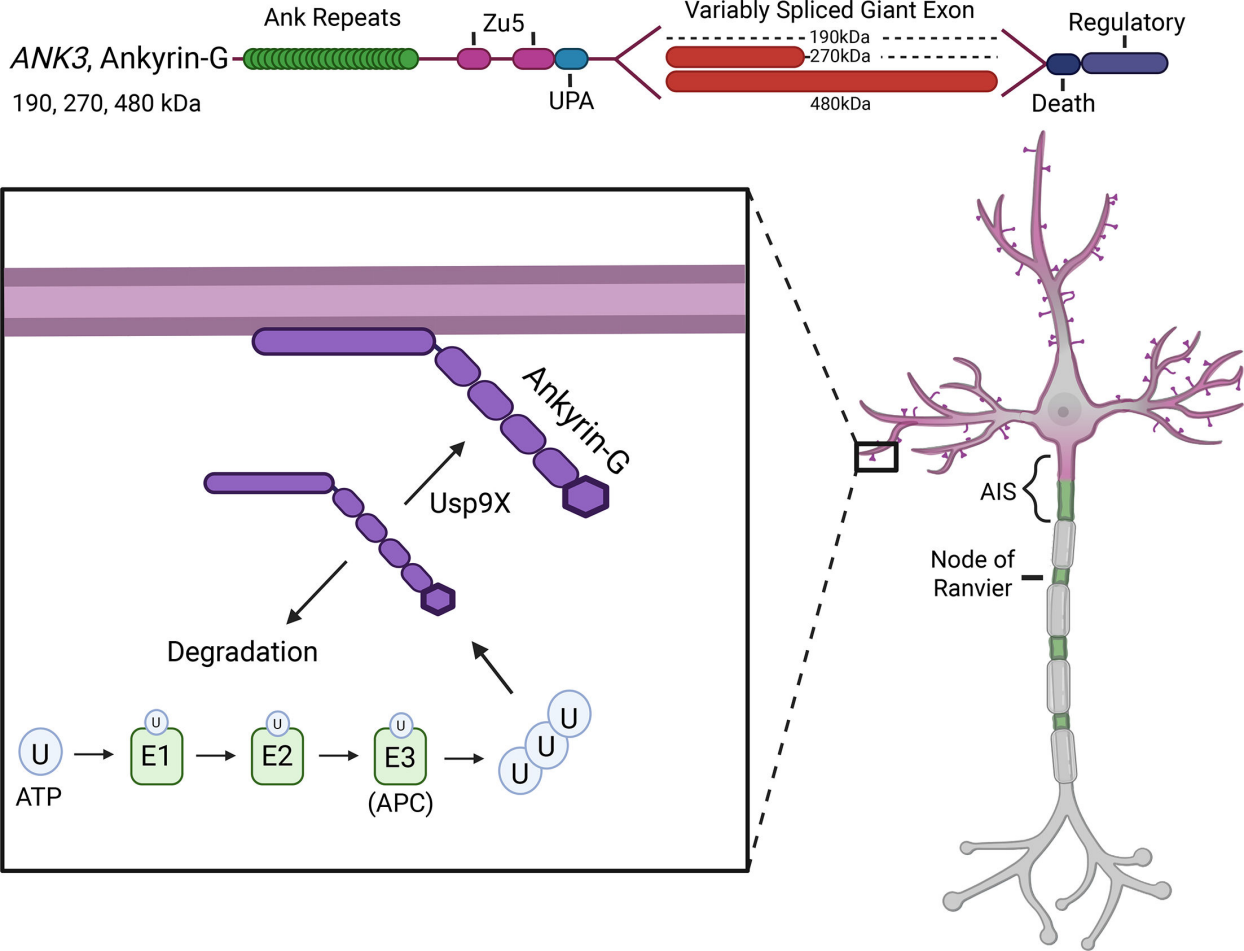
Ankyrin-G ubiquitination. (Top) Diagram of 190 kDa ankyrin-G. (Bottom) 190 kDa ankyrin-G is ubiquitinated to promote degradation, while deubiquitination by Usp9X stabilizes ankyrin-G at dendritic spines.

Ubiquitin modifications can also be removed from proteins by deubiquitinating enzymes, and this process may play a role in the regulation of gene expression [79]. Because of this, disruption of ubiquitination could be a key cause of an array of developmental diseases [81]. One deubiquitinating enzyme that is implicated in neurodevelopmental disability, Usp9X, was recently found to regulate ankyrin-G at synapses [75]. Usp9X is responsible for decreased ankyrin-G polyubiquitination and is critical in the stabilization of ankyrin-G for proper maintenance of dendritic spine morphology [75](Figure 5). A lack of Usp9X in mouse cortex also revealed a decrease in spine density and an increase in ankyrin-G aggregates, which illustrates the importance of this enzyme in normal ankyrin-G function at the dendrites [75]. Compellingly, ankyrin-B levels were also found to be increased without the presence of Usp9X, which suggests that both ankyrins are regulated by the ubiquitin system, and possibly by the same machinery [75]. Clearly, the intricate balance of ankyrin ubiquitination is critical for its protein levels and function in the maintenance of dendritic spines and synaptic plasticity.

### Conclusions

Overall, the proper localization of ankyrins is essential to normal neuronal structure and function, and post-translational modifications are a key regulator of this. Future studies will shed more light on how ankyrins are localized throughout neuronal development and what role post-translational modifications play in this. More work needs to be done to understand how and why ankyrins are post-translationally modified and how this fits into the context of neurological disorders. Along with this, characterization of post-translational modifications of ankyrin binding partners could give further insight into ankyrin localization. Interestingly, several ankyrin binding partners have been found to undergo post-translational modifications [57,82,83]. There is emerging evidence that concomitant modification of proteins can aid in complex assembly, and this may be the case for ankyrins and the proteins they scaffold. Additionally, the identification of the full enzymatic machinery involved in modification of ankyrins would be useful in the context of disease. Due to the reversibility of phosphorylation, palmitoylation, and ubiquitination, the enzymes that regulate ankyrin modifications make intriguing drug targets. Potential up- or down-regulation of ankyrin modifications could be useful in the treatment of neurological diseases implicated in VGSC and ankyrin dysfunction.

**Perspectives**

- Ankyrins are scaffolding proteins that must be properly localized for normal neuronal function. Ankyrin dysfunction is linked to multiple neurological disorders such as epilepsy and autism spectrum disorder.
- Subcellular localization of ankyrins changes throughout neuronal development so that they can complete distinct roles.
- Ankyrins are post-translationally modified to regulate protein localization and overall neuronal function. Regulation of post-translational modifications affects ankyrin trafficking and interaction with binding partners.

## Abbreviations

AIS: axon initial segment
ASD: autism spectrum disorder
CAM: cell adhesion molecule
CK2: casein kinase 2
PPT1: palmitoyl protein thioesterase 1
VGSC: voltage-gated sodium channel
